# Comparison of Quantitative and Qualitative EDXRF Analysis for Provenance Study of Archaeological Ceramics

**DOI:** 10.3390/ma17153725

**Published:** 2024-07-27

**Authors:** Maja Gajic-Kvascev, Velibor Andric, Radmila Jancic-Heinemann, Ognjen Mladenovic, Aleksandar Bulatovic

**Affiliations:** 1 Department of Chemical Dynamics and Permanent Education, Vinča Institute of Nuclear Sciences, National Institute of the Republic of Serbia, University of Belgrade, 11000 Belgrade, Serbia; 2 Faculty of Technology and Metallurgy, University of Belgrade, 11000 Belgrade, Serbia; 3 Institute of Archaeology, National Institute of the Republic of Serbia, 11000 Belgrade, Serbia

**Keywords:** archaeological ceramics, clay, EDXRF spectrometry, pattern recognition, classification

## Abstract

The most common scientific analysis of archaeological ceramics aims to determine the raw material source and/or production technology. Scientists and archaeologists widely use XRF-based techniques as a tool in a provenance study. After conducting XRF analysis, the results are often analyzed using multivariate analysis in addition to interpretation and conclusions. Various multivariate techniques have already been applied in archaeological ceramics provenance studies to reveal different raw material sources, identify imported pieces, or determine different production recipes. This study aims to evaluate the results of multivariate analysis in the provenance study of ceramics that belong to three cultures that settled in the same area during various prehistoric periods. Portable energy-dispersive X-ray fluorescence spectrometry (pEDXRF) was used to determine the elemental composition of the ceramic material. The ceramic material was prepared in two different ways. The ceramic body material was ground into powder, homogenized, and then pressed into tablets. After that, the same fragments are polished in suitable places. Quantitative and qualitative analyses were performed on the tablets and polished pieces. The results were subjected to both unsupervised and supervised multivariate analysis. Based on the results, it was concluded that qualitative analysis of the well-prepared shards’ surface using EDXRF spectrometry could be utilized in provenance studies, even when the ceramic assemblages were made of similar raw materials.

## 1. Introduction

The production of ceramic in prehistory can be considered state-of-the-art technology. Understanding this process provides valuable insights into the skills of the manufacturers, trading patterns, and overall development of early civilizations [1]. Since ceramics do not change over time, analyzing the materials provides reliable conclusions about whether they were made from local raw materials or resulted from trade with others. Scientific examining archaeological ceramics usually involves various analytical techniques to reveal their chemical and/or mineralogical composition [2,3,4,5]. Nowadays, these analyses can be conducted either by sampling the ceramic materials or in a non-invasive manner [6,7]. Moreover, provenance studies can be performed using both quantitative and qualitative analytical results [8,9,10].

**Figure 1:**
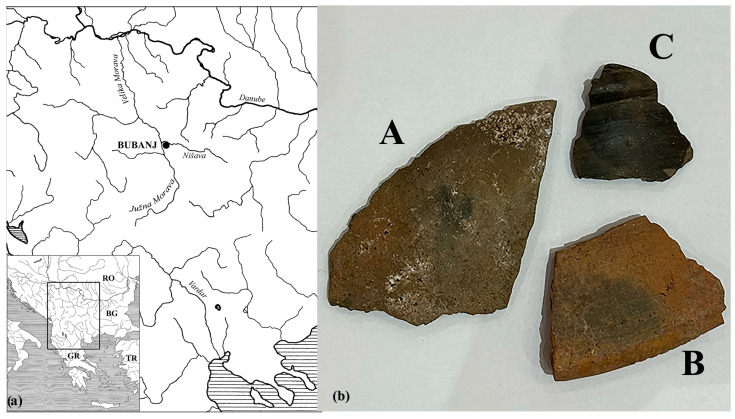
(**a**) Location of the site of Bubanj, Serbia (drawing: A. Bulatović); (**b**) Representative ceramic fragments from the Bubanj Hum I (A), Cernavoda III (B) and Coţofeni–Kostolac (C) culture.

Many documented provenance studies have focused on differentiating between ceramic assemblages or identifying imported ceramics. Different material characteristics, such as elemental, chemical, or mineralogical composition, were analyzed using pattern recognition techniques to draw conclusions about variations in raw materials and/or production technology among ceramic findings. The present study aims to evaluate the effectiveness of various pattern recognition techniques used for the provenance study of archaeological ceramics from different prehistoric cultures that were made of similar raw materials. For that purpose, archaeologists carefully selected and grouped the ceramic fragments according to stratigraphy, typology, and dating. They chose a suitable site where three cultures settled in prehistory for a relatively short period. The Bubanj archaeological site is in the southern Pomoravlje region, in the central part of the Niš Valley, approximately 7 km southeast of the confluence of the Nišava and South Morava rivers (today’s Serbia, Figure 1a). Early excavations in this area (which started in the 1930s) revealed multilayered stratigraphy. This site was inhabited in the Middle Neolithic period, with the earliest findings associated with the Starcevo culture (in the first half of the 6th millennium BC). The Eneolithic and early Bronze Age layers follow mentioned cultural layers, dated to the Middle Neolithic [11]. The first cultural layer in this study belongs to the so-called Bubanj Hum I culture, corresponding to the Early Eneolithic and being a regional representative of the widely spread Bubanj–Salcuţa–Krivodol cultural complex. The findings from the upper layer, from the Middle Eneolithic, belong to Cernavoda III, with elements of Baden and Boleraz cultures. The latest group included in this study is from the Late Eneolithic period and belongs to the Coţofeni–Kostolac cultural layer. All three layers were well dated to the corresponding period and cultures [12]. The ceramic assemblages were formed from fragments that belong to each cultural layer. According to archaeologists, Bubanj Hum I culture settled on this site for the longest time, implying that local raw materials were used for ceramic production. The Cernavoda III settled the site long after Bubanj Hum I culture; therefore, they could not take over previous knowledge but rather found local clay sources nearby the site. Only the fragments from the Coţofeni–Kostolac cultural layer might have been brought to the site during the settlement. Those archaeological assumptions were tested in this study to evaluate analytical and multivariate protocols for the provenience study.

The ceramic material was analyzed using EDXRF spectrometry. The quantitative and qualitative analytical results were subjected to dimension reduction to evaluate suitability for classification. The most-used unsupervised and supervised techniques were chosen for the evaluation [13,14,15]. These techniques were evaluated based on several parameters to measure their efficiency in reducing dimensions and their ability to separate different classes, which is crucial for designing classifiers. The classification’s effectiveness was evaluated by considering its recognition ability and its rate of misclassification.

This study aims to examine how the method of preparing archaeological ceramics for analysis affects classification efficiency. For this purpose, samples were taken from the material of the selected fragments from which tablets were made. Their elemental composition was quantified using standard reference material (CRM). The elemental composition was also determined for the analyses performed on well-polished places on the fragments. The qualitative and quantitative results obtained in this way are classified using different dimension reduction techniques. The selection of ceramic materials from different archaeological stratigraphies, made from similar raw materials, is such that it does not prejudice an effective classification. Based on the achieved results, qualitative analytical results can be used in provenience studies as effectively as quantitative ones.

## 2. Materials and Methods

### 2.1. Sample Description

The ceramic fragments are grouped according to the stratigraphy layers in which they were found, and special attention was paid to selecting the fragments from reliable archaeological contexts corresponding to each cultural layer with certainty. The first group, labeled BI, contains 18 fragments belonging to the so-called Bubanj Hum I culture and comes from a structure (pit) marked as 69. The second one, denoted as CV, contains 18 pottery vessel shards (structure 108-pit) from the upper layer, considered Cernavoda III. The last group, labeled as KK, is formed of the 13 shards that belong to the Coţofeni–Kostolac cultural layer. These shards were taken from the oldest (first) phase found on the house floor (structure 15), which can be considered brought in during settlement. Macroscopic archaeological analysis indicates similarities between the findings from the BI and CV ceramic fragments but differences with the KK group (Figure 1b).

The ceramic fragments were prepared for analysis in two different ways. Small amounts of the ceramic material were powdered by grinding the ceramic body from its various parts. A handy sander equipped with a diamond blade was used. The fragments were cleaned before grinding. The obtained ceramic powder was fine for tableting, which was performed without prior sieving. The homogenized material was then pressed into large tablets, each 3 cm in diameter, containing an equal amount of the material. After that, suitable parts of the fragments were polished and cleaned. Each fragment was analyzed at three points, and the average values were used for further analysis [16]. To quantify the chemical composition, the IAEA PT ancient Chinese ceramic certificated reference material (CRM, [17]) was tableted in the same manner as the ceramic samples and analyzed under the same conditions. Three clay samples from nearby clay pits were also analyzed under the same conditions as the ceramic material to test the assumption about local raw material sources. The nearest clay pit, Crepana, is about 800 m west–southwest of Bubanj. This pit was used until the 1940s. Today, it is closed but easily accessible for sampling. The Tri Bresta clay pit is 2 km from the site in the same direction, which also worked until the middle of the last century. The farthest clay deposit, Crepana near Lalinac, is located 2.6 km west of the Bubanj site and remains active. All three clay deposits are located at a 15–35 min walk from the site on flat surroundings, so the probability of their exploitation in the Eneolithic is high.

### 2.2. Analytical Technique and Datasets Forming

The EDXRF spectrometry was employed for quantitative and qualitative elemental analysis of the ceramic material using an in-house-developed milli-beam spot instrument. The spectrometer consists of an air-cooled X-ray tube (Oxford Instruments, Scotts Valley, CA, USA, Rh anode, maximum voltage 50 kV, maximum current 1 mA) with a pin-hole collimator and a Si-PIN X-ray detector (6 mm2/500 m, Be window 12.5 m thickness, with energy resolution of 160 eV at 5.89 keV), associated with a DSP (X123, Amptek Inc., Bedford, MA, USA) for spectra acquisition. Two laser pointers were used for the accurate sample, and tablet positioning. ADMCA software (Amptek Inc., version 1, 0, 0, 16)) was used for spectra acquisition and processing. The following parameters: X-ray tube voltage of 35 kV and 800 μA current, without filter, were kept constant during all measurements and measuring time of 120 s.

An original MATLAB code was developed to align the EDXRF spectra using the peaks balancing procedure to minimize any experimental setup contributions. All EDXRF spectra were aligned before further analysis. The dimension reduction procedure was performed on raw spectra containing overall spectral information along with the datasets containing characteristic elemental composition. This was done to examine the influence of the feature selection process, which involves potentially losing some information about the dataset structure. The feature selection was performed by quantifying the chemical composition using CRM and using a radial-basis neural network (RBNN) procedure. The EDXRF results were quantified using the Net Peak Area parameter. This parameter was determined by ADMCA software by marking the peak area. For each EDXRF spectrum, the Net Peak Area parameter was calculated for nine peaks, corresponding to the elements determined in the CRM. These nine values can be considered as selected features, and their informativeness for classification purposes will be evaluated in this study. The data selection using RBNN was performed as another suitable feature selection method. The detailed procedure is described in [18]. The RBNN was designed and trained to reach maximal reconstruction of the initial EDXRF spectrum using normal distribution functions. The maximal height parameter of the nine functions was utilized to characterize ceramic fragment material. The procedure is used because it is much faster and more reliable than calculating the Net Peak Area parameter, so it is suitable for testing.

### 2.3. Pattern Recognition Techniques

Principal component analysis (PCA) was selected among unsupervised dimension reduction techniques as it is widely used in analyzing cultural heritage objects [19,20]. PCA creates a reduced space of maximal variance, where the first few components account for most of the variation in the original datasets. Based on the above definition, applying PCA to the provenience studies of ceramic materials made from different raw materials will result in considerable classification possibilities. In this study, the classification possibilities of the PCA will be evaluated for the dataset of ceramics made of the same or similar raw material.

PCA may not be able to accurately represent the dataset’s group membership due to the small variance expected. Therefore, a supervised method called scattering matrices-based dimension reduction was tested, aiming to preserve group coherence as much as possible during dimension reduction. More mathematical details about PCA and scattering matrices-based dimension reduction can be found in [21,22].

To determine which dimension reduction technique is more effective for provenance studies, it is important to understand how much of the initial dataset structure is preserved during the dimension reduction transformation. The effectiveness of PCA transformation to lower dimensional space is quantified by the percentage of the dataset’s total variance preserved along the projection axes [23]. The amount of information lost during dimension reduction is measured by the index of informativeness, a parameter defined as:

(1) $$l_{d/n}=\frac{\sum_{i=1}^{d}\lambda_{i}}{\sum_{j=1}^{n}\lambda_{j}}$$

where *λ_i_*—eigenvalues of the covariance matrix, set in descending order, *d*—dimension of reduced space, and *n*—dimension of initial space. The higher value of this parameter indicates that dimension reduction was performed in a way that minimized information loss. The Bhattacharyya distance *μ*(1/2) (a measure of separability between groups in the space of reduced dimensions) is defined as [24]:

(2) $$\mu (1/2)=\frac{1}{8}{\left(M_{2}-M_{1}\right)}^{T}{\left[\frac{\Sigma_{1}+\Sigma_{2}}{2}\right]}^{-1}(M_{2}-M_{1})+\frac{1}{2}\ln\frac{\left|\frac{\Sigma_{1}+\Sigma_{2}}{2}\right|}{\sqrt{\left|\Sigma_{1}\right|\left|\Sigma_{2}\right|}}$$

where *M*_1_ and *M*_2_ denote expected vectors and Σ_1_ and Σ_2_ denote covariance matrices. A larger parameter value indicates that two classes are separable, and a space between them is large enough for classifier design. The *μ*(1/2) parameter value will be discussed according to the possibility of linear classifier design. The recognition ability parameter (defined as the percentage of correctly classified dataset members and mathematically represented by the ratio of correctly classified and the total number of dataset members) was calculated to evaluate the linear classification effectiveness, together with the percentage of the misclassified results.

## 3. Results and Discussion

### 3.1. EDXRF Spectrometry Analysis

**Figure 2:**
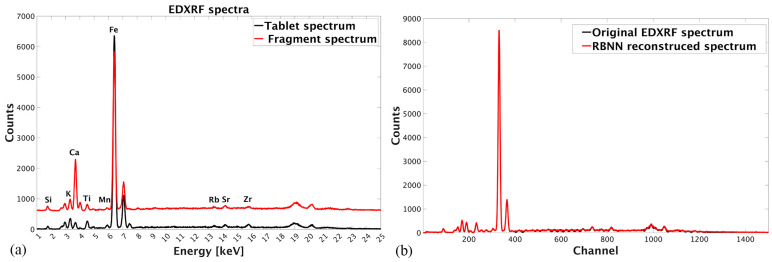
(**a**) EDXRF spectra aligned using original spectra alignment procedure; (**b**) Radial basis neural network spectra reconstruction for feature selection.

According to the collected EDXRF spectra (Figure 2a), Si, K, Ca, Ti, Mn, Fe, Rb, Sr, and Zr were detected in both the tablets and ceramic fragments. The same chemical composition contains the tablet of the CRM, which was used to quantify ceramics’ chemical composition. The raw EDXRF spectral data collected for both tablets and ceramic fragments, aligned prior, were reduced to exclude non-informative channels before Si and after Zr. The data were organized in a matrix with the dimensions 36 × 1700, denoted as RT and RF, for tablets and fragments, respectively. Further, RBNN was employed for feature selection from raw spectra. A comparison of the original and RBNN reconstructed spectra is presented in Figure 2b. The reconstruction enabled total superposition of the spectra and high confidence in the data selection. The RBNN was used to extract the maximum value of the most distinctive peak for the same nine chemical elements from the EDXRF spectra. Using the described procedure, two more datasets were organized as a matrix with the dimension of 36 × 9 for both fragments (denoted as NF) and tablets (denoted as NT). The Net Peak Area parameter was used to quantify the tablets’ elemental composition; the results are presented in Table 1. The dataset organized in the matrix with dimensions of 36 × 9 was denoted as QT.

All the experimental data subjected to PCA and SMB dimension reduction were autoscaled before analysis.

### 3.2. Dimension Reduction and Classification Results

**Figure 3:**
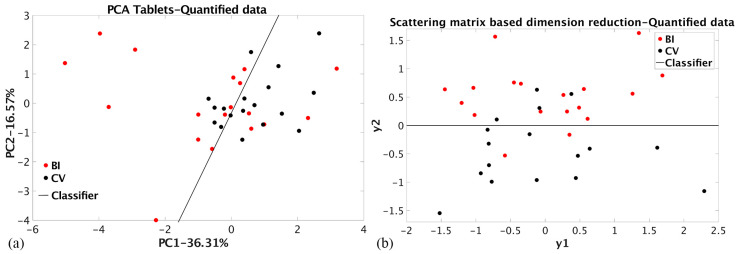
(**a**) PCA dimension reduction of the tablet’s quantified elemental composition (QT dataset); (**b**) SMB dimension reduction of the tablet’s quantified elemental composition (QT dataset).

The dimension reduction results of a dataset containing elemental composition quantified values (QT) can be considered the reference because the analysis was performed on homogenized material, and the exact composition is determined. The results of the PCA dimension reduction of the QT dataset are presented in Figure 3a. As can be seen, the between-class distance and group coherence are small, with 52.88% of the total variance preserved. The Bhattacharya distance value is 0.31. Nevertheless, the linear classifier is designed with a recognition ability of nearly 36% for the BI dataset and 33% for the CV. The classification enabled the misclassification of 28% of BI and 33% of CV samples. The supervised SMB dimension reduction of the same datasets achieved slightly higher between-class distance, with the Bhattacharya distance value of 0.39. At the same time, group coherence remained small in the new y1–y2 space (Figure 3b). The linear classification parameters (together with errors given in brackets) are shown in Table 2. Supervised SMB dimension reduction enables only 11% of BI and 22% of CV samples to be misclassified

**Figure 4:**
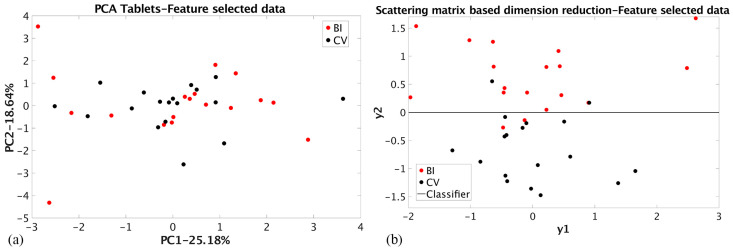
(**a**) PCA dimension reduction of the tablet’s RBNN selected elemental composition (NT dataset); (**b**) SMB dimension reduction of the tablet’s RBNN selected elemental composition (NT dataset).

**Figure 5:**
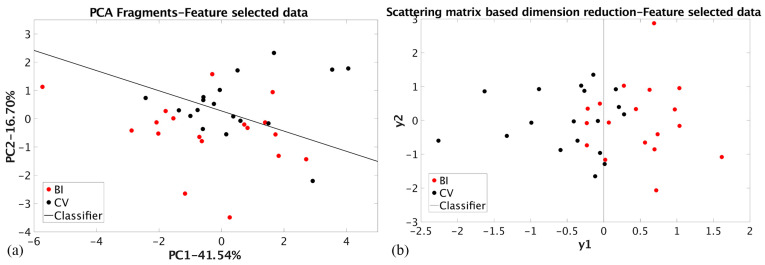
(**a**) PCA dimension reduction of the ceramic fragment’s RBNN selected elemental composition (NF dataset); (**b**) SMB dimension reduction of the ceramic fragment’s RBNN selected elemental composition (NF dataset).

The results achieved using nine peak maximum values determined for tablets by the RBNN selection procedure (NT dataset, Figure 4) are shown in Table 2. The PCA dimension reduction showed that linear classification is impossible, even though 43.82% of the total variance was preserved. The group coherence is also small, which can be explained by the fact that some grouping information was lost by data selection. The SMB dimension reduction of the same dataset enabled linear classification with the highest value of the Bhattacharya distance (Table 2) and the smallest percentage of the misclassified samples, 11% for both groups. For comparison, the PCA and SMB dimension reduction results for the RBNN-selected data for the ceramic fragments (NF dataset) are shown in Figure 5. For both cases, linear classification is possible. The PCA achieved the highest value of the total variance preserved (58.24%) with a relatively small value of the Bhattacharya distance (0.16).

**Figure 6:**
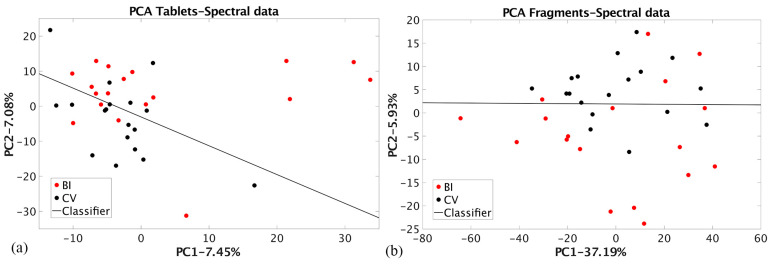
PCA dimension reduction of the raw EDXRF spectral data for (**a**) tablets (RT dataset) and (**b**) ceramic fragments (RF dataset).

The designed classifier enabled misclassification of 17% of BI and 50% of CV samples. The SMB dimension reduction achieved a slightly higher misclassification of 17% for BI and 22% for CV samples compared to the results for the tablet’s dataset. The comparison of SMB techniques on the two datasets with selected features favors the NT dataset over the QT dataset (*μ*(1/2): 0.39 vs. 0.70, RA: 44% for BI and both techniques and 39% vs. 44% for CV, MC: 11% for BI and both techniques and 22% vs. 11% for CV). This is because part of the information about the dataset structure is lost during the quantification process, likely due to feature selection and an ancient porcelain standard. The results of the PCA dimension reduction of the raw EDXRF spectral data for tablets (RT dataset) and ceramic fragments (RF dataset) are shown in Figure 6.

The linear classification is possible using raw spectral data even if the l_d/n_ parameter values are small (Table 2). More confident results are achieved for the fragments dataset in the PCA space, where less structural data were lost during the dimension reduction. Group coherence is smaller for the fragments dataset, resulting in a smaller Bhattacharya distance value. The classification parameters have the same values (Table 2). This result is highly significant as it shows that the dimension reduction of the dataset containing the qualitative results of the fragment analysis achieves classification results comparable to those of quantitative results on the homogenized tablets. This implies that a provenance study based on qualitative EDXRF results can be conducted with high reliability.

The small group coherence and the Bhattacharya distance values can be explained by using the same/similar clay sources for ceramic production, resulting in a small variance between data (Figure S1 in Supplementary Materials). The linear classifier was still possible to design, likely due to changes in production technology, such as adding different tempering materials.

**Figure 7:**
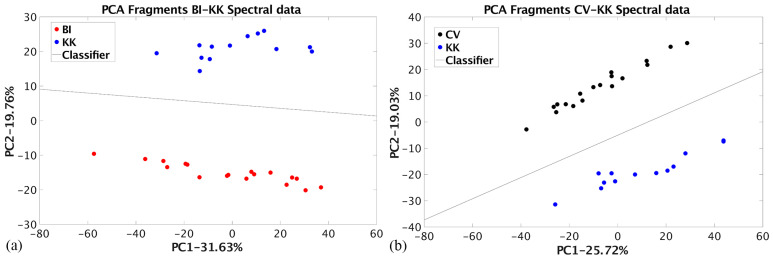
PCA dimension reduction of the raw EDXRF spectral data (**a**) for Bubanj Hum I and Coţofeni–Kostolac dataset; (**b**) for Cernavoda III and Coţofeni–Kostolac dataset.

The raw EDXRF spectral data obtained for Coţofeni–Kostolac ceramic fragments assemblages were organized in a matrix with a dimension of 13 × 1700. This dataset was subjected to PCA-based dimension reduction with Bubanj Hum I and Cernavoda III fragments. The analysis was performed to compare the results of dimension reduction and classification between the sets of non-similar raw materials (Figures S2 and S3 in Supplementary Materials). The results are shown in Figure 7.

The percentage of the total variance preserved in the PC1-PC2 space is 51.39% for the BI-KK and 44.75% for the CV-KK datasets. These results are comparable to those presented in Table 2. The Bhattacharya distance values are 20.37 and 27.27, respectively. These values suggest that classification can be performed without any misclassification.

## 4. Conclusions

The effectiveness of the two most-used dimension reduction techniques, PCA as an unsupervised method and SMB as a supervised method, was evaluated for the archaeological ceramic provenance study. In this evaluation, both quantitative and qualitative EDXRF analytical results were used. The chemical composition of the sampled ceramic materials, homogenized and pressed into tablets, was quantified using CRM and used as referent classification. An RBNN-based procedure was also used to determine the same chemical composition faster and more reliably to test the amount of lost information during feature extraction. Qualitative analysis using EDXRF spectrometry conducted on tablets and ceramic shards aimed to compare their effectiveness in the provenance study among themselves and with quantitative results. The dimension reduction was assessed based on the percentage of preserved total variance and the possibility of linear classification. The Bhattacharya distance was calculated in the reduced space to measure between-class separability as an indicator for effective classification. The recognition ability and percentage of misclassification were calculated for linear classification evaluation.

The preserved total variance ranged from 14.5% to 58%, providing a less representative picture of the initial dataset structure in the reduced space. This result was followed by small group coherence in the reduced spaces and low values of the Bhattacharya distance. Even so, it was possible to design the linear classifier, and the classification results showed a small misclassification. The parameters used to evaluate the success of dimension reduction and classification have similar values for quantitative and qualitative results, which is the most important result (l_d/n_: 52.88% vs. 43.12%, *μ*(1/2): 0.31 vs. 0.25, RA: 36% vs. 39% for BI and 33% vs. 36% for CV, MC: 28% vs. 22% for BI and 33% vs. 28% for CV). Although quantification leads to precise chemical composition, it was shown that it might not always be the most informative method for provenance study and comparison with other assemblages, as some important information may be lost during feature selection. The qualitative analysis of the well-prepared shards’ surface using EDXRF spectrometry can be utilized in provenance studies, even when the ceramic assemblages were made of similar raw materials. The results indicate that qualitative EDXRF results can be used reliably for provenance studies of archaeological ceramics, similar to the quantitative ones. This method requires easier sample preparation, is much faster, and does not necessitate the use of CRM.

## Figures and Tables

**Table 1:** Chemical composition of the ceramic materials from BI and CV ensemble.

	SiO_2_, %	K_2_O, %	CaO, %	TiO_2_, %	MnO, %	Fe_2_O_3_, %	Rb, ppm	Sr, ppm	Zr, ppm
BI1	86.82	3.98	1.29	0.86	0.05	5.30	50.22	94.51	153.59
BI2	69.51	3.29	2.56	0.79	0.04	5.48	66.84	95.19	169.15
BI3	78.77	3.42	2.11	0.96	0.05	5.11	65.82	60.53	144.78
BI4	90.31	3.26	2.12	0.80	0.05	4.95	80.41	113.33	152.69
BI5	71.66	3.54	8.73	0.52	0.03	3.63	72.47	111.26	170.44
BI6	79.44	2.65	12.21	0.45	0.03	3.11	63.24	128.71	109.27
BI7	71.39	3.31	2.10	0.76	0.06	5.67	52.53	107.67	128.58
BI8	54.35	2.85	11.11	0.43	0.04	2.96	88.07	97.80	159.04
BI9	67.77	3.61	9.30	0.52	0.03	3.67	50.59	102.62	104.73
BI10	60.12	3.58	2.45	0.83	0.04	5.12	61.12	101.93	111.60
BI11	72.47	4.41	1.91	0.72	0.04	4.64	61.21	116.85	111.86
BI12	75.95	3.35	1.98	0.76	0.04	5.43	66.38	68.34	182.37
BI13	85.62	3.13	1.39	0.72	0.05	4.66	95.92	80.20	142.19
BI14	68.71	3.56	2.21	0.83	0.05	5.33	80.60	98.87	132.60
BI15	77.70	2.84	1.91	1.14	0.05	6.12	72.84	64.51	202.07
BI16	88.30	3.56	2.04	0.70	0.04	4.58	73.21	74.69	167.85
BI17	55.96	1.14	6.57	0.54	0.06	7.12	45.98	68.11	91.77
BI18	84.01	3.78	1.19	0.80	0.13	5.31	90.20	61.22	215.03
CV1	78.91	3.84	2.16	0.83	0.03	5.59	63.89	108.66	152.95
CV2	69.51	4.97	2.44	0.94	0.07	6.50	73.39	76.06	152.17
CV3	88.84	4.14	2.51	0.70	0.10	5.19	78.38	97.41	203.50
CV4	97.56	4.68	2.49	0.96	0.07	6.13	84.38	87.31	149.84
CV5	80.11	4.25	2.27	0.81	0.08	5.56	87.70	100.63	145.69
CV6	83.47	3.84	3.48	0.85	0.05	4.34	81.61	92.90	197.02
CV7	73.67	4.24	2.20	0.86	0.06	6.02	77.73	92.29	121.19
CV8	86.02	4.34	2.21	0.81	0.07	5.10	58.53	109.58	142.84
CV9	85.48	3.90	2.17	0.88	0.07	5.30	71.18	66.42	143.74
CV10	75.95	3.84	1.96	0.71	0.05	4.82	61.39	91.75	120.67
CV11	72.20	4.31	2.61	0.72	0.11	4.88	68.69	89.53	147.89
CV12	70.05	3.59	1.83	0.83	0.03	4.58	64.07	64.05	197.02
CV13	87.09	4.07	7.95	0.71	0.04	5.10	63.52	80.50	146.34
CV14	53.54	4.38	2.21	0.66	0.06	5.64	78.93	92.98	114.19
CV15	75.42	3.02	2.09	0.70	0.05	5.27	82.35	99.86	117.17
CV16	58.91	3.80	3.35	0.65	0.08	5.15	75.43	88.23	150.09
CV17	87.50	4.41	3.37	0.69	0.09	5.88	59.64	97.34	100.58
CV18	69.92	3.72	1.72	1.08	0.05	5.25	98.14	87.62	277.12
SRM-CC	67.50	2.30	0.62	0.95	0.03	2.70	113.00	103.00	337.00

**Table 2:** Dimension reduction and classification parameters.

Material	Dimension Reduction	Dataset	l_d/n_ (%)	*μ*(1/2)	BI Samples		CV Samples	
					RA *	MC *	RA	MC
Tablets	PCA	Quantified (QT)	52.88 (2.37)	0.31 (0.007)	36	28	33	33
		RBNN feature selection (NT)	43.82 (2.54)	0.08 (0.003)	*	*	*	*
		Full spectra (RT)	14.54 (0.86)	0.33 (0.009)	39	22	36	28
	SMB	Quantified (QT)	100 (0.81)	0.39 (0.022)	44	11	39	22
		RBNN feature selection (NT)	100 (0.72)	0.70 (0.006)	44	11	44	11
Fragments	PCA	RBNN feature selection (NF)	58.24 (2.97)	0.16 (0.008)	42	17	25	50
		Full spectra (RF)	43.12 (1.08)	0.25 (0.007)	39	22	36	28
	SMB	RBNN feature selection (NF)	100 (0.95)	0.35 (0.003)	42	17	39	22

* RA—Recognition ability (%), MC—Misclassification (%).

## Data Availability

Data are contained within the article and supplementary materials.

## Supplementary Materials

The following supporting information can be downloaded at: https://www.mdpi.com/article/10.3390/ma17153725/s1, Figure S1: PCA dimension reduction of the BI and CV ceramic fragments datasets using raw EDXRF spectral data. The clay material was classified using the linear classifier. Figure S2: PCA dimension reduction of the BI and KK ceramic fragments datasets using raw EDXRF spectral data. The clay material was classified using the linear classifier. Figure S3: PCA dimension reduction of the CV and KK ceramic fragments datasets using raw EDXRF spectral data. The clay material was classified using the linear classifier.
