# Supplementary material for: Comparison of Quantitative and Qualitative EDXRF Analysis for Provenance Study of Archaeological Ceramics
Source: Materials (Basel). 2024 Jul 27;17(15):3725. doi: 10.3390/ma17153725 (PMC11313242; doi:10.3390/ma17153725)
Supplement: Supplementary file 1 [file materials-17-03725-s001.zip › materials-3088169-supplementary.pdf]

Supplementary Materials

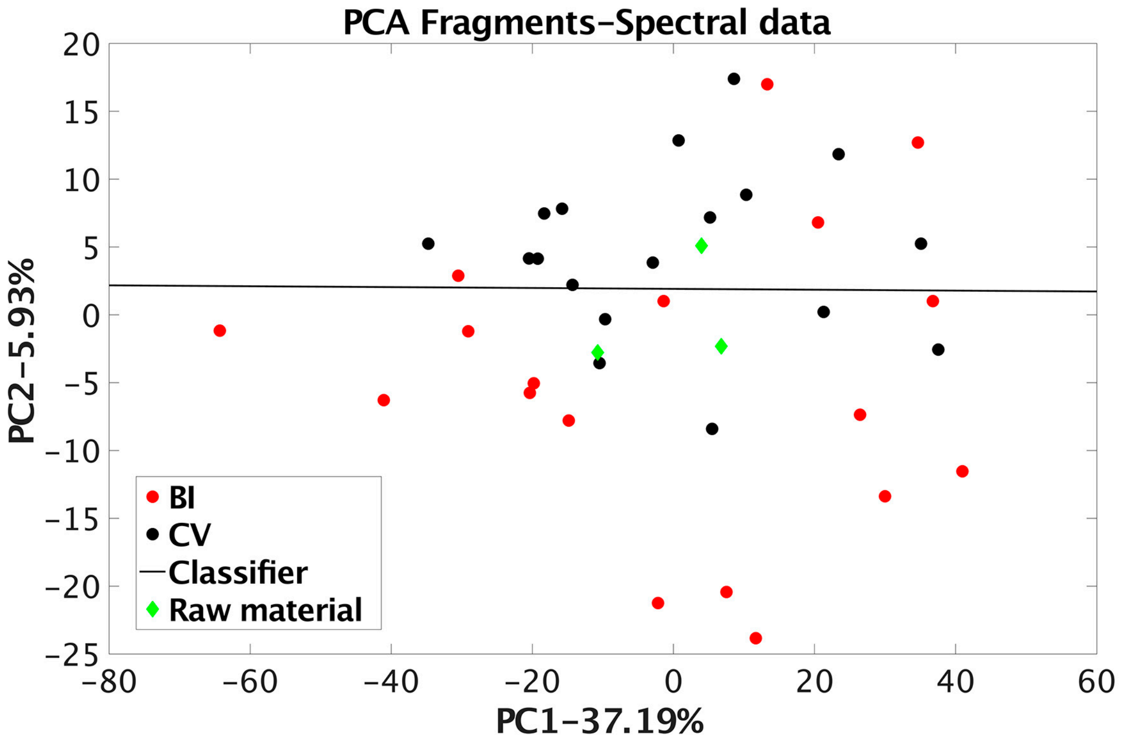

Figure S1. PCA dimension reduction of the BI and CV ceramic fragments datasets using raw EDXRF spectral data. The clay material was classified using the linear classifier.

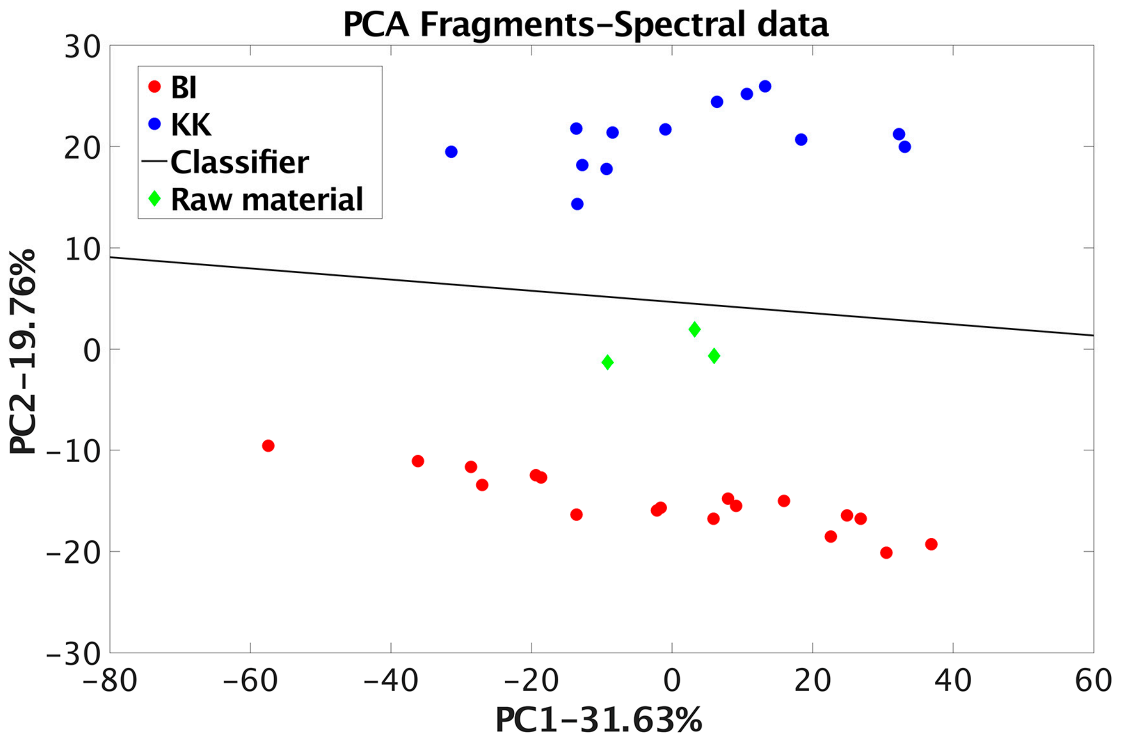

Figure S2. PCA dimension reduction of the BI and KK ceramic fragments datasets using raw EDXRF spectral data. The clay material was classified using the linear classifier.

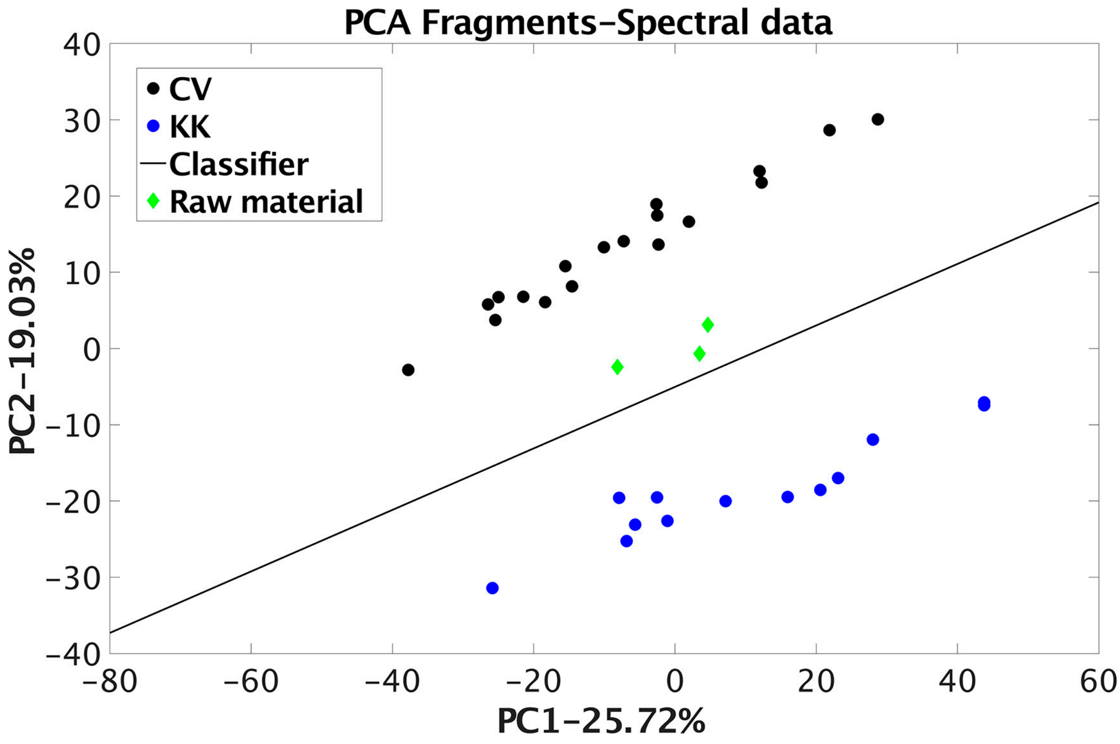

Figure S3. PCA dimension reduction of the CV and KK ceramic fragments datasets using raw EDXRF spectral data. The clay material was classified using the linear classifier.
